# Supplementary material for: Probing the gating mechanism of the mechanosensitive channel Piezo1 with the small molecule Yoda1
Source: Nat Commun. 2018 May 23;9:2029. doi: 10.1038/s41467-018-04405-3 (PMC5966384; doi:10.1038/s41467-018-04405-3)
Supplement: Supplementary file 1 — Supplementary Information [file 41467_2018_4405_MOESM1_ESM.pdf]

Probing the Gating Mechanism of the Mechanosensitive Channel Piezo1 with the Small Molecule Yoda1

Lacroix et al.

Supplementary Figures

```
                                          F1961
hPZ1   E-KRPSRSGGRVRAAGRRLQGFCLSLAQGTYRPLRRFFHDILHTKYRAATDVYALMFLADVVDFIIIIFGFWAFGKHS
mPZ1   HTQEKSKFRERMKAAGRRLQSFCVSLAQSFYQPLQRFFHDILHTKYRAATDVYALMFLADIVDIIIIIFGFWAFGKHS
hPZ2   -----ELYMEKLQEHLIKAKAFTIKKTLEIYVPIKQFFYNLIHPEYSAVTDVYVLMFLADTVDFIIIVFGFWAFGKHS
mPZ2   -----ELYMEKLQEHLIKAKAFTIKKTLQIYVPIRQFFYDLIHPDYSAVTDVYVLMFLADTVDFIIIVFGFWAFGKHS
                                             * ** ** * *    *      *      *

      T2005                          R2035                       F2063
hPZ1   AATDITSSLSDDQVPEAFLVMLLIQFSTMVVDRALYLRKTVLGKLAFQVALVLAIHLWMFFILPAVTERMFNQNVVAQ
mPZ1   AATDIASSLSDDQVPQAFLFMLLVQFGTMVIDRALYLRKTVLGKLAFQVVLVVAIHIWMFFILPAVTERMFSQNAVAQ
hPZ2   AAADITSSLSEDQVPGPFLVMVLIQFGTMVVDRALYLRKTVLGKVIFQVILVFGIHFWMFFILPGVTERKFSQNLVAQ
mPZ2   AAADITSSLSEDQVPGPFLVMVLIQFGTMVVDRALYLRKTVLGKVIFQVILVFGIHFWMFFILPGVTERKFSQNLVAQ
         *       *    **    *                      **   *  **  *

hPZ1   LWYFVKCIYFALSAYQIRCGYPTRILGNFLTKKYNHLNLFLFQGFRLVPFLVELRAVMDWVWTDTTLSLSSWMCVEDI
mPZ1   LWYFVKCIYFALSAYQIRCGYPTRILGNFLTKKYNHLNLFLFQGFRLVPFLVELRAVMDWVWTDTTLSLSNWMCVEDI
hPZ2   LWYFVKCVYFGLSAYQIRCGYPTRVLGNFLTKSYNYVNLFLFQGFRLVPFLTELRAVMDWVWTDTTLSLSSWICVEDI
mPZ2   LWYFVKCVYFGLSAYQIRCGYPTRVLGNFLTKSYNYVNLFLFQGFRLVPFLTELRAVMDWVWTDTTLSLSSWICVEDI

hPZ1   YANIFIIKCSRETEKKYPQPKGQKKKKIVKYGMGGLIILFLIAIIWFPLLFMSLVRSVVGVVNQPIDVTVTLKLGGYE
mPZ1   YANIFIIKCSRETEKKYPQPKGQKKKKIVKYGMGGLIILFLIAIIWFPLLFMSLIRSVVGVVNQPIDVTVTLKLGGYE
hPZ2   YAHIFILKCWRESEKRYPQPRGQKKKKVVKYGMGGMIIVLLICIVWFPLLFMSLIKSVAGVINQPLDVSVTITLGGYQ
mPZ2   YAHIFILKCWRESEKRYPQPRGQKKKKAVKYGMGGMIIVLLICIVWFPLLFMSLIKSVAGVINQPLDVSVTITLGGYQ
                                                                  P2456
hPZ1   PLFTMSAQQPSIIPFTAQAYEELSRQFDPQPLAMQFISQYSPEDIVTAQIEGSSGALWRISPPSRAQMKRELYNGTAD
mPZ1   PLFTMSAQQPSIVPFTPQAYEELSQQFDPYPLAMQFISQYSPEDIVTAQIEGSSGALWRISPPSRAQMKQELYNGTAD
hPZ2   PIFTMSAQQSQLKVMDQQSFNKFIQAFSRDTGAMQFLENYEKEDITVAELEGNSNSLWTISPPSKQKMIHELLDPNSS
mPZ2   PIFTMSAQQSQLKVMDNSKYNEFLKSFGPNSGAMQFLENYEREDVTVAELEGNSNSLWTISPPSKQKMIQELTDPNSC

hPZ1   ITLRFTWNFQRDLAKGGTVEYANEKHMLALAPNSTARRQLASLLEGTSDQ----SVVIPNLFPKYIRAPNGPEANPVK
mPZ1   ITLRFTWNFQRDLAKGGTVEYTNEKHTLELAPNSTARRQLAQLLEGRPDQ----SVVIPHLFPKYIRAPNGPEANPVK
hPZ2   FSVVFSWSIQRNLSLGAKSEIATDKLSFPLK--NITRKNIAKMIAGNSTESSKTPVTIEKIYPYYVKAPSDSNSKPIK
mPZ2   FSVVFSWSIQRNMTLGAKAEIATDKLSFPLA--VATRNSIAKMIAGNDTESSNTPVTIEKIYPYYVKAPSDSNSKPIK

hPZ1   QLQPNEEADYLGVRIQLRREQGA----------GATGFLEWWVIELQECR---TDCNLLPMVIFSDKVSPPSLGFLAG
mPZ1   QLQPDEEEDYLGVRIQLRREQVGTGASGEQAGTKASDFLEWWVIELQDCK---ADCNLLPMVIFSDKVSPPSLGFLAG
hPZ2   QLLS--ENNFMDITIILSRDNTTKY------------NSEWWVLNLTGNRIYNPNSQALELVVFNDKVSPPSLGFLAG
mPZ2   QLLS--ENNFMNITIILFRDNVTKS------------NSEWWVLNLTGSRIFNQGSQALELVVFNDKVSPPSLGFLAG

hPZ1   YGIMGLYVSIVLVIGKFVRGFFSEISHSIMFEELPCVDRILKLCQDIFLVRETRELELEEELYAKLIFLYRSPETMIK
mPZ1   YGIVGLYVSIVLVVGKFVRGFFSEISHSIMFEELPCVDRILKLCQDIFLVRETRELELEEELYAKLIFLYRSPETMIK
hPZ2   YGIMGLYASVVLVIGKFVREFFSGISHSIMFEELPNVDRILKLCTDIFLVRETGELELEEDLYAKLIFLYRSPETMIK
mPZ2   YGIMGLYASVVLVIGKFVREFFSGISHSIMFEELPNVDRILKLCTDIFLVRETGELELEEDLYAKLIFLYRSPETMIK

hPZ1   WTREKE-
mPZ1   WTRERE-
hPZ2   WTREKTN
mPZ2   WTREKTN
```

Supplementary Figure 1: Sequence alignment (T-coffee) of the C-terminal region of mouse Piezo1 (mPZ1), Piezo2 (mPZ2) and human Piezo1 (hPZ1) and Piezo2 (hPZ2). The residues that are numbered and shaded correspond to positions used to create the chimeras and sub-domain chimeras. Asterisks indicate residues within the 1961-2063 region that are overall not conserved between Piezo1 and Piezo2.

Supplementary Tables

Supplementary Table 1: Fitted parameters for the I / Imax vs. Pressure plots using equation 1

	mPZ1	mPZ1 + 30 µM Yoda1	Chim	Chim + 30 µM Yoda1
k (mmHg)	9.46 ± 0.64	10.78 ± 1.51	5.73 ± 0.47	5.95 ± 0.48
P_{50} (mmHg)	-52 ± 1	-26 ± 1	-62 ± 1	-63 ± 1
R^2	0.988	0.952	0.980	0.979

Supplementary Table 2: Fitted parameters for different gating models

Number of activated subunits needed for pore opening		≥ 1	≥ 2	3
fitting equation from main text		2	3	4
Syeda et al.	B_{max}	2069.85 ± 100.69	1818.63 ± 48.79	1836.90 ± 55.18
	K_d (µM)	22.92 ± 2.95	10.83 ± 0.76	4.13 ± 0.32
	R^2	0.993	0.996	0.995
this study	B_{max}	1.67 ± 0.07	1.50 ± 0.08	1.49 ± 0.08
	K_d (µM)	10.92 ± 1.44	5.20 ± 0.85	1.89 ± 0.32
	R^2	0.980	0.954	0.948

Supplementary Table 3: Fitted parameters of I / I_{max} plots for WT and WT:Chim = 1:1 in presence of varying Yoda1 concentrations using equation 1.

	WT			WT:Chim = 1:1		
[Yoda1] (µM)	k (mmHg)	P_{50} (mmHg)	R^2	k (mmHg)	P_{50} (mmHg)	R^2
1	10.2 ± 0.7	-44.4 ± 0.8	0.996	9.3 ± 1.0	-40 ± 1.1	0.991
3	10.4 ± 0.7	-36.4 ± 0.8	0.988	9.9 ± 1.1	-36 ± 0.8	0.991
10	10.9 ± 0.7	-31.2 ± 0.8	0.988	9.6 ± 1.1	-27 ± 0.8	0.956
30	10.3 ± 0.7	-25.7 ± 0.7	0.985	11.3 ± 1.3	-20 ± 0.6	0.908
100	6.9 ± 0.6	-16.9 ± 0.6	0.976	13 ± 1.3	-18 ± 0.9	0.940

Supplementary Note 1

The measured fluorescence F is a function of the saturation function ν of the protein P by the agonist ligand L, of the background fluorescence q and of a constant b_{max}:

$$F = q + (b_{\max}\,\nu)\ (1)$$

Before addition of the ligand (t = 0), ν = 0 and thus $F_0 = q$. The relative change of fluorescence follows:

$$\frac{F - F_0}{F_0} = \frac{(q + (b_{\max}\,\nu)) - q}{q} = \frac{b_{\max}}{q}\ \nu = B_{\max}\,\nu \quad (2)$$

with $B_{max} = b_{max} / q$. In case of multiple binding sites per protein, the saturation function equals the total concentration of bound ligand ($[L]_{bound}$) over the total concentration of protein ($[P]_0$):

$$\nu = \frac{[L]_{\text{bound}}}{[P]_0} \quad (3)$$

Assuming each subunit of the trimeric channel interacts independently with the agonist:

$$\frac{[L]_{\text{bound}}}{[P]_0} = \frac{[PL] + 2[P\mathrm{L}_2] + 3[P\mathrm{L}_3]}{[P] + [PL] + [P\mathrm{L}_2] + [PL_3]} \quad (4)$$

The different protein species are related by the corresponding macroscopic dissociation constant K_1, K_2 and K_3 corresponding to each binding step:

$$K_1 = \frac{[P][L]}{[PL]}\ ;\ K_2 = \frac{[PL][L]}{[PL_2]}\ ;\ \ K_3 = \frac{[PL_2][L]}{[PL_3]}$$

By replacing the macroscopic constants:

$$\nu\ = \frac{\frac{[L]}{K_1} + \frac{2[L]}{K_1K_2} + \frac{3[L]}{K_1K_2K_3}}{1 + \frac{[L]}{K_1} + \frac{[L]}{K_1K_2} + \frac{[L]}{K_1K_2K_3}} \quad (5)$$

The macroscopic constants are related to the microscopic dissociation constant K_d by the number of possible binding combinations $\Omega_{n,i}$ for each binding step i and for n binding sites:

$$K_i = \frac{\Omega_{n,i-1}}{\Omega_{n,i}} K_{\text{d}} = \frac{\binom{n}{i-1}}{\binom{n}{i}} = \frac{i}{n - i + 1}\ K_{\text{d}}$$

Replacing the macroscopic constants by the K_d gives and using $n = 3$ for a trimeric channel:

$$\nu = \frac{\frac{3[L]}{K_\mathrm{d}} + \frac{6[L]^2}{{K_\mathrm{d}}^2} + \frac{3[L]^3}{{K_\mathrm{d}}^3}}{1 + \frac{3[L]}{K_\mathrm{d}} + \frac{3[L]^2}{{K_\mathrm{d}}^2} + \frac{[L]^3}{{K_\mathrm{d}}^3}} \quad (6)$$

Supplementary Equation 6 further simplifies by applying a binomial reduction:

$$\nu = \frac{3[L]}{K_\mathrm{d} + [L]} \quad (7)$$

Assuming the activation of one or more subunit is sufficient for channel opening, every species in the numerator of Supplementary Equation 6 contribute to the observed fluorescence signal:

$$\frac{F - F_0}{F_0} = B_\mathrm{max} \frac{3[L]}{K_\mathrm{d} + [L]} \quad (8)$$

However, if the activation of two or three subunits is required for channel activation, the fraction of channel with a single activated subunit does not contribute to the signal. In this case, the fluorescence signal follows:

$$\frac{F - F_0}{F_0} = B_\mathrm{max} \frac{\frac{6[L]^2}{{K_\mathrm{d}}^2} + \frac{3[L]^3}{{K_\mathrm{d}}^3}}{1 + \frac{3[L]}{K_\mathrm{d}} + \frac{3[L]^2}{{K_\mathrm{d}}^2} + \frac{[L]^3}{{K_\mathrm{d}}^3}} \quad (9)$$

Similarly, if the activation of three subunits is necessary for channel activation, the fluorescence signal follows:

$$\frac{F - F_0}{F_0} = B_\mathrm{max} \frac{\frac{3[L]^3}{{K_\mathrm{d}}^3}}{1 + \frac{3[L]}{K_\mathrm{d}} + \frac{3[L]^2}{{K_\mathrm{d}}^2} + \frac{[L]^3}{{K_\mathrm{d}}^3}} \quad (10)$$
