# Supplementary material for: Probing the gating mechanism of the mechanosensitive channel Piezo1 with the small molecule Yoda1
Source: Nat Commun. 2018 May 23;9:2029. doi: 10.1038/s41467-018-04405-3 (PMC5966384; doi:10.1038/s41467-018-04405-3)
Supplement: Supplementary file 3 — Description of Additional Supplementary Files [file 41467_2018_4405_MOESM3_ESM.pdf]

Description of Additional Supplementary Files

File Name: Supplementary Movie 1
Description: Time-lapse calcium imaging of Yoda1-mediated Piezo1 activation. HEK293T cells co-expressing GCaMP6m and mPiezo1 were imaged for one minute at 1 frame s-1 under epifluorescence illumination. 100 µM Yoda1 was added at t = 10 s. The images are displayed in the movie at 5 frames s-1.

File Name: Supplementary Movie 2
Description: Yoda1-mediated calcium signals are Piezo1-dependent. HEK293T cells expressing GCaMP6m were imaged for one minute at 1 frame s-1 under epifluorescence illumination. 100 µM Yoda1 was added at t = 10 s. The images are displayed in the movie at 5 frames s-1.
